# Supplementary material for: Comparing multiscale, presence-only habitat suitability models created with structured survey data and community science data for a rare warbler species at the southern range margin
Source: PLoS One. 2023 Apr 12;18(4):e0275556. doi: 10.1371/journal.pone.0275556 (PMC10096272; doi:10.1371/journal.pone.0275556)
Supplement: S1 Table — (PDF) [file pone.0275556.s001.pdf]

Table S1. Data sources for landcover and topographic variables included in Golden-winged Warbler Maxent habitat distribution models.

Variable	Data Source	Year(s)	Original Data Type	Original Resolution
Forest height 0-10m within 150m	LANDFIRE Existing Vegetation Height https://landfire.gov/evh.php	2008, 2012, 2014	Categorical	30x30m
Forest height 25-50m within 150m	LANDFIRE Existing Vegetation Height https://landfire.gov/evh.php	2008, 2012, 2014	Categorical	30x30m
Road cover within 150m	LANDFIRE Existing Vegetation Height https://landfire.gov/evh.php	2001, 2008, 2012, 2014, 2016, 2020	Categorical	30x30m
Forest height 25-50m within 2500m	LANDFIRE Existing Vegetation Height https://landfire.gov/evh.php	2008, 2012, 2014	Categorical	30x30m
Road cover within 2500m	LANDFIRE Existing Vegetation Height https://landfire.gov/evh.php	2001, 2008, 2012, 2014, 2016, 2020	Categorical	30x30m
Agricultural land within 2500m	LANDFIRE Existing Vegetation Height https://landfire.gov/evh.php	2001, 2008, 2012, 2014, 2016, 2020	Categorical	30x30m
Developed land within 2500m	LANDFIRE Existing Vegetation Height https://landfire.gov/evh.php	2001, 2008, 2012, 2014, 2016, 2020	Categorical	30x30m
Herb and shrub cover within 150m	Rangeland Analysis Platform Vegetation Cover V3 http://rangeland.ntsg.umt.edu/data/rap/rap-vegetation-cover/	2000-2020 (21 years)	Continuous (Percent)	30x30m
Herb and shrub cover within 2500m	Rangeland Analysis Platform Vegetation Cover V3 http://rangeland.ntsg.umt.edu/data/rap/rap-vegetation-cover/	2000-2020 (21 years)	Continuous (Percent)	30x30m
Canopy cover within 150m	NLCD USFS Canopy Cover https://www.mrlc.gov/data	2011, 2016	Continuous (Percent)	30x30m
Canopy cover within 2500m	NLCD USFS Canopy Cover https://www.mrlc.gov/data	2011, 2016	Continuous (Percent)	30x30m
Elevation	ASTER Global DEM V3 https://earthexplorer.usgs.gov/	2019	Continuous (Percent)	30x30m
Slope	ASTER Global DEM V3 https://earthexplorer.usgs.gov/	2019	Continuous (Percent)	30x30m
Aspect	ASTER Global Digital Elevation Model V3 https://earthexplorer.usgs.gov/	2019	Continuous (Percent)	30x30m
