# Supplementary material for: Comparing multiscale, presence-only habitat suitability models created with structured survey data and community science data for a rare warbler species at the southern range margin
Source: PLoS One. 2023 Apr 12;18(4):e0275556. doi: 10.1371/journal.pone.0275556 (PMC10096272; doi:10.1371/journal.pone.0275556)
Supplement: S2 Table — (PDF) [file pone.0275556.s002.pdf]

Table S2. Ecological niche description including mean values (± standard deviation) for all variables included in Maxent models.

Variable	Audubon	eBird	Background
Forest height 0-10m within 150m	0.02 ± 0.04	0.02 ± 0.04	0.008 ± 0.03
Forest height 25-50m within 150m	0.14 ± 0.14	0.08 ± 0.12	0.21 ± 0.18
Road cover within 150m	0.05 ± 0.06	0.08 ± 0.09	0.03 ± 0.06
Forest height 25-50m within 2500m	0.24 ± 0.07	0.22 ± 0.08	0.25 ± 0.10
Agricultural land within 2500m	0.001 ± 0.002	0.002 ± 0.006	0.003 ± 0.007
Developed land within 2500m	0.0008 ± 0.002	0.005 ± 0.02	0.004 ± 0.01
Herb and shrub cover within 150m	0.16 ± 0.16	0.17 ± 0.17	0.10 ± 0.15
Herb and shrub cover within 2500m	0.10 ± 0.06	0.10 ± 0.06	0.10 ± 0.08
Elevation	1097 ± 186	1128 ± 302	968 ± 250
Slope	14.6 ± 7.0	12.8 ± 6.2	16.6 ± 8.5
Aspect	3.0 ± 1.8	3.2 ± 1.7	3.2 ± 1.8
