# Supplementary material for: Comparing multiscale, presence-only habitat suitability models created with structured survey data and community science data for a rare warbler species at the southern range margin
Source: PLoS One. 2023 Apr 12;18(4):e0275556. doi: 10.1371/journal.pone.0275556 (PMC10096272; doi:10.1371/journal.pone.0275556)
Supplement: Attachment — Submitted filename: PONE-D-22-25984.docx [file pone.0275556.s006.docx]

Comparing multiscale, presence-only habitat suitability models created with structured

survey data and community science data for a rare warbler species at the southern

range margin

The topic of this research can be found interesting in the bird conservation community as it targets a rare warbler species. Authors used 2 different datasets to compare their ability to predict habitat suitability of the species. The manuscript needs to be revised in both aim and goals and methods. My main comment is about using GLM for presence-background points. It is showed by various studies that machine learning methods specifically Maxent are much better than GLM for presence background points. Authors might find following comments useful for their manuscript.

Line 71: In which regions these were conducted? I agree that species in different regions might have different habitat requirements. Please explain a little more this concept based on, for instance, evolutionary processes to justify this gap of knowledge. Also, there are some other warblers showing multi-scale habitat selection than can be mentioned here. For example, please see: Amirkhiz *et al.* Investigating niches and distribution of a rare species in a hierarchical framework: Virginia’s Warbler (*Leiothlypis virginiae*) at its northeastern range limit. *Landscape Ecol* **36**, 1039–1054 (2021). <https://doi.org/10.1007/s10980-021-01217-7>

Line 91. It was difficult for me to understand how studying habitat associations and genetic studies are related. This knowledge gap needs to be explained better to justify this study.

Lines 107-118: If this is a knowledge gap which this study is based upon, authors need to compare current survey area to what they found as “potential suitable habitats” and then recommend explicit management actions.

Lines 122-127: This is not really what ebird data are. have structured data gathered based on specific protocols to meet specific goals and standards. Please review this reference: <https://cornelllabofornithology.github.io/ebird-best-practices/>

Lines 1330-135: This justification needs to be reconsidered. Ebird data are structured data and heavily wetted by experts. They are not just some points. Please see the above reference.

Line 140. Authors mention in the introduction that the lack of knowledge on differences between habitat associations of this species in their study area and other areas is a justification for conducting their study so their goal needs to be accordingly.

Line 147: please explain what this focal area is

Line 150: Please add state lines to the U.S map. Also add breeding range and the focal area mentioned above

Line 153: One of the main benefits of ebird data is having absence points. Why did not authors use them in their study? Also, ebird data has strong standards to reduce the impact of sampling bias. Did authors follow ebird standard process?

It seems Audobon NC and ebird data have been gathered under very similar conditions. One of the goals of this study is to compare these 2 datasets. Thus, it is necessary to explain, in introduction, why this is an important research question? What are differences and how these differences can affect ecological studies or management actions. These can be explained as hypotheses or research questions.

Line 166: why 100 meters?

Line 173: How about climatic and topographic variables? If this study is all about associations with landcover data, goals and objectives should be restricted accordingly, assuming other habitat factors are constant or have no associations. Also, using only 2014 LC data for a dataset covering 200-2020 is based on the assumption that landcover did not change during this period. However, authors, as a reason for conducting this research, mentioned in introduction that habitat loss and human development are 2 main reasons for reductions in this species population. I would suggest to either revising goals and objectives or using NLCD data which has a finer temporal resolution. If the latter, please use closest NLCD layer for each year and extract corresponding landcover data for each point. Also, please check cropland data layers and <https://www.ntsg.umt.edu/project/landsat/landsat-landcover.php> . Vegetation height of land fire data still can be used along with NLCD or other landcover datasets.

Line 178: Which package? Citation. This comment applies to all methods and techniques.

Lines 180-185: based on what assumptions these buffers and mesasures were selected?

Line 186: how many presence points for each dataset?

Line 189: There are many papers proving that Maxent or other Machine learning methods have better performance for presence-only data. Merrow et al 2014 do not recommend using GLM for modeling habitat associations. They provide a range of options based on goals and the nature of data. Based on Merrow and many other papers I believe Maxent or any other machine learning methods are better fit for these data. The main reason is using only presence data. I would consider GLM as an appropriate method if authors used absence data as well. please see the following reference:

Guisan, A., Thuiller, W., & Zimmermann, N. (2017). Habitat Suitability and Distribution Models: With Applications in R (Ecology, Biodiversity and Conservation). Cambridge: Cambridge University Press. doi:10.1017/9781139028271

Also, if investigating habitat associations is the main goal of this study, creating and interpretation of response curves should be a part of the paper. So, I strongly recommend including response curves in this study.

Please use more metrics for evaluating models if comparing 2 different datasets is the goal. Each metrics have advantages and limitations. Please see Guisan et al 2017.
