# Supplementary material for: Comparing multiscale, presence-only habitat suitability models created with structured survey data and community science data for a rare warbler species at the southern range margin
Source: PLoS One. 2023 Apr 12;18(4):e0275556. doi: 10.1371/journal.pone.0275556 (PMC10096272; doi:10.1371/journal.pone.0275556)
Supplement: Attachment — Submitted filename: Response to Comments.docx [file pone.0275556.s007.docx]

Comparing multiscale, presence-only habitat suitability models created with structured

survey data and community science data for a rare warbler species at the southern

range margin

Reviewer #1: The topic of this research can be found interesting in the bird conservation community as it targets a rare warbler species. Authors used 2 different datasets to compare their ability to predict habitat suitability of the species. The manuscript needs to be revised in both aim and goals and methods. My main comment is about using GLM for presence-background points. It is showed by various studies that machine learning methods specifically Maxent are much better than GLM for presence background points. Authors might find following comments useful for their manuscript.

Reviewer #1, thank you for your detailed response and helpful comments. We have revised our manuscript to address your concerns, including a reanalysis of the data using Maxent. Please see revised methods and results, and the responses to comments below.

Line 71: In which regions these were conducted? I agree that species in different regions might have different habitat requirements. Please explain a little more this concept based on, for instance, evolutionary processes to justify this gap of knowledge. Also, there are some other warblers showing multi-scale habitat selection than can be mentioned here. For example, please see: Amirkhiz *et al.* Investigating niches and distribution of a rare species in a hierarchical framework: Virginia’s Warbler (*Leiothlypis virginiae*) at its northeastern range limit. *Landscape Ecol* **36**, 1039–1054 (2021). <https://doi.org/10.1007/s10980-021-01217-7>

We added citations for other bird species with multi-scale habitat selection (Line 76). We also added the regions where most Golden-winged Warbler research is conducted (Lines 85-87). We discuss evolutionary processes that justify why populations in different regions may have different habitat preferences in Lines 80-85.

Line 91. It was difficult for me to understand how studying habitat associations and genetic studies are related. This knowledge gap needs to be explained better to justify this study.

That is understandable. We have removed discussion of genetic studies (around line 90) from our introduction, as we do not believe it was background needed to justify a study on habitat associations.

Lines 107-118: If this is a knowledge gap which this study is based upon, authors need to compare current survey area to what they found as “potential suitable habitats” and then recommend explicit management actions.

We point out now in the discussion that GWWA in Western North Carolina are likely not using early successional habitat that is only herb/shrub, but rather a mix of young trees and herb/shrub. We recommend in our discussion that survey efforts focus on smaller, more structurally complex patches of early successional habitat (Lines 437-448).

Lines 122-127: This is not really what ebird data are. have structured data gathered based on specific protocols to meet specific goals and standards. Please review this reference: <https://cornelllabofornithology.github.io/ebird-best-practices/>

We have revised our description of eBird data to describe it as “semi-structured”, as it is referred to in the eBird best practices document. We have also added a more detailed description of the playback protocol used by Audubon NC, which is one of the primary differences between the datasets (eBird users are not using this 20-min long standardized playback protocol). See lines 132-152.

Lines 1330-135: This justification needs to be reconsidered. Ebird data are structured data and heavily wetted by experts. They are not just some points. Please see the above reference.

We have revised Lines 126-152 to address this concern. We now emphasize that Audubon NC surveys are conducted with the use of a standardized playback protocol and compare and contrast the datasets in lines 132-152.

Line 140. Authors mention in the introduction that the lack of knowledge on differences between habitat associations of this species in their study area and other areas is a justification for conducting their study so their goal needs to be accordingly.

Thank you – we have added phrasing to goal (1) in Line 152 to incorporate the goal of understanding habitat associations in WNC compared to other areas in the breeding range.

Line 147: please explain what this focal area is

Removed. These focal areas were defined by the Golden-winged Warbler Working Group as the remaining areas after the range contraction of the last 50 years. The breeding range covers the same area, so we removed mention of the focal areas since it is redundant.

Line 150: Please add state lines to the U.S map. Also add breeding range and the focal area mentioned above

Added state lines to the US map and clarified in the caption that the breeding range in WNC as defined by the US Geological Survey is the same as the study area. Basically, we used the publicly available USGS breeding range and clipped it by the North Carolina state boundary to get our study area. We removed mention of the focal areas for simplicity – this is not really needed for our study.

Line 153: One of the main benefits of ebird data is having absence points. Why did not authors use them in their study? Also, ebird data has strong standards to reduce the impact of sampling bias. Did authors follow ebird standard process?

Great questions – we now include a discussion of why we chose to use presence-background in our analysis (Lines 181-190). We followed the eBird best practices (Strimas-Mackey et al. 2020) by filtering out traveling checklists and studying landcover associations within buffers around presence points to account for some spatial noise. eBird best practices do not require you to use only complete checklists unless you are inferring absences.

It seems Audobon NC and ebird data have been gathered under very similar conditions. One of the goals of this study is to compare these 2 datasets. Thus, it is necessary to explain, in introduction, why this is an important research question? What are differences and how these differences can affect ecological studies or management actions. These can be explained as hypotheses or research questions.

We now explain in more detail how eBird and Audubon data collection efforts differ in Lines 132-152.

Line 166: why 100 meters?

We now include a description of why we use 100m as our buffer distance for data redundancy (Lines 197-199). We used 100m because it is the low end of the maximum detection distance for Golden-winged Warblers in mixed shrub/forest habitats, and because it is the median radius of territory size identified in the GWWG conservation plan.

Line 173: How about climatic and topographic variables? If this study is all about associations with landcover data, goals and objectives should be restricted accordingly, assuming other habitat factors are constant or have no associations. Also, using only 2014 LC data for a dataset covering 200-2020 is based on the assumption that landcover did not change during this period. However, authors, as a reason for conducting this research, mentioned in introduction that habitat loss and human development are 2 main reasons for reductions in this species population. I would suggest to either revising goals and objectives or using NLCD data which has a finer temporal resolution. If the latter, please use closest NLCD layer for each year and extract corresponding landcover data for each point. Also, please check cropland data layers and <https://www.ntsg.umt.edu/project/landsat/landsat-landcover.php> . Vegetation height of land fire data still can be used along with NLCD or other landcover datasets.

Thank you for these suggestions. We have now included higher temporal resolution for all variables by using all available datasets from the years 2000-2020 (see Supplementary Material Appendix S1). We now use the Rangeland Analysis Platform datasets to describe herb/shrub cover, which seems to work much better than the LandFire dataset we were using before (thank you for the recommendation). We also now include topographic variables elevation, slope, and aspect derived from ASTER DEM data. We chose to not use climatic variables because we were primarily interested in understanding landcover and topographic associations for Golden-winged Warbler management, and climate variables also likely correlate with topographic and landcover variables.

Line 178: Which package? Citation. This comment applies to all methods and techniques.

Added citations for all R packages (raster, dismo, sp, and ENMeval).

Lines 180-185: based on what assumptions these buffers and mesasures were selected?

We now include a discussion of why buffers and variables were selected in lines 230-237.

Line 186: how many presence points for each dataset?

Included in the paper, lines 206-207, 253, 363-364.

Line 189: There are many papers proving that Maxent or other Machine learning methods have better performance for presence-only data. Merrow et al 2014 do not recommend using GLM for modeling habitat associations. They provide a range of options based on goals and the nature of data. Based on Merrow and many other papers I believe Maxent or any other machine learning methods are better fit for these data. The main reason is using only presence data. I would consider GLM as an appropriate method if authors used absence data as well. please see the following reference:

Guisan, A., Thuiller, W., & Zimmermann, N. (2017). Habitat Suitability and Distribution Models: With Applications in R (Ecology, Biodiversity and Conservation). Cambridge: Cambridge University Press. doi:10.1017/9781139028271

Thank you for the advice on revising the methods – we have now reanalyzed the data using Maxent. Please see new methods and results.

Also, if investigating habitat associations is the main goal of this study, creating and interpretation of response curves should be a part of the paper. So, I strongly recommend including response curves in this study.

Great point – we now include Maxent response curves as one of our main figures in the paper (Fig 2).

Please use more metrics for evaluating models if comparing 2 different datasets is the goal. Each metrics have advantages and limitations. Please see Guisan et al 2017.

We now compare datasets using variable importance values, response curves, and AUC values. We also extract prediction values from one dataset at presence points for the opposite dataset and calculate and compare AUC values for those cross-dataset predictions. AUC is the primary evaluation metric used in Maxent model performance.

Reviewer #2: Lines 37 - 40: The second goal of the paper is not clear in the abstract (to determine if community science data produces similar distribution models as systematic sampling data).

We now include an explicit mention of the second goal of the paper in the abstract (Lines 37-40)

Line 47. I suggest: Additionally “our results help to validate the use of bird data, since they produce similar species distribution modeling results…”

Added, thank you.

Methods

We have now reanalyzed the data using Maxent, based on comments from Reviewer #1. Please see revised methods and results for details.

Method needs to specify the M used to model and if it was the same for eBird and Audubon NC data. Since it is well known that administrative divisions are not a good option. I suppose you used an M based on ecological characteristics relevant to the species.

Both models were created with the same methods, see revised methods and results for details.

You clearly state USFS land-fire raster resolution, however, It is not clear if EVH and NLCD rasters were already at 30m x 30m pixel size or if the resolution was changed.

We now explicitly mention the original pixel size of all rasters and include this information in the Supplementary Material (Table S1).

Method also needs to specify how many of the presence points were used for training models and testing models in each case (eBird and Audubon NC data)

Total presence points are included in the paper, lines 206-207, 253, 363-364. In the methods, we specify that we use 10-fold cross-validation (Lines 274-278), where we set aside 10% of the data for testing, using 90% for training, repeating this 10x until all presence points have been used for both training and testing.

I think you should consider using ku.enm (Cobos et al. 2019) for the process, starting from model calibration. Among other benefits, ku.enm evaluates model performance using partial ROC, instead of area under the ROC curve, that has been prove to be a a more suitable indicator of statistical significance.

Since we reran the analysis using Maxent, we now use the ENMeval package (Kass et al. 2021) for model tuning, model performance metrics, etc.

I wonder if there is information in eBird data to use only confirmed breeding presence, since I understand Audubon NC data are only confirmed breeding data. If I understood correctly a more thorough selection of eBird data could provide an even more similar model. If this is not the case you could at least discuss this in the corresponding section.

eBird has a way to specify on a checklist whether a bird was observed doing different breeding behaviors, including singing, sitting on a nest, feeding chicks, etc. This would be a way you could “confirm” breeding – if you see a bird on a nest, witness a copulation, or witness a feeding. However, few eBird users submit these observations, and it would vastly decrease the number of points in the dataset (originally downloaded eBird observations: 1572, after filtering for confirmed breeding: 9). This is a great idea though, and hopefully more checklists will start to include these breeding bird codes. Notably, the Audubon NC data are not necessarily confirmed breeding data, but they are territorial responses by (mostly) males to playback, which makes it a very likely breeding territory. So, there is slightly more confidence in the Audubon NC dataset representing breeding sites, but not necessarily confirmed unless a nest is found. I added some of this information to the discussion, Lines 466-479.

I understand the resolution you used somehow prevents you from using other environmental data such as temperature, however maybe you could have considered to use lidar data for topographic variables.

We now include topographic variables elevation, slope and aspect, calculated from ASTER DEM data.

Even when you explain the importance of vegetation height for the species, I wonder if the use of a limited set of variables could be overestimating the importance of the variables when describing the niche.

We added more variables, including both landcover and topographic, to address this issue. We also include variable importance measures from the Maxent models showing which variables contribute the most to the models.

I suggest to include a table describing the ecological niche. A table with means and SD of each variable for every model (Audubon and eBird)

We now include a table describing the ecological niche in the Supplementary Material (Table S1-2), including means and standard deviations for Audubon, eBird, and background datasets.

The title is more focused on model comparison, however, the abstract and the discussion seem more focused on the importance of ENM proper description for conservation. I suggest to try to include both goals in the title.

We want to emphasize model comparison in the title – we have revised the abstract and discussion to shift the focus back to model comparison.
